# Must egalitarians oppose private healthcare?

**DOI:** 10.1007/s11019-026-10365-z

**Published:** 2026-05-25

**Authors:** Johann Go

**Affiliations:** Global Business School for Health, University College London (UCL), Marshgate Building, 7 Sidings Street, London, E20 2AE UK

**Keywords:** Egalitarianism, Private healthcare, Health equity, Health justice, Liberalism

## Abstract

A central tenet of health equity is that access to healthcare should be responsive to need rather than patients’ socioeconomic status. The idea that ability to pay can enable some people to access faster, better, or more healthcare strikes many egalitarians as clearly objectionable. This paper argues that this view is more complicated than may initially appear. While there are egalitarian reasons to oppose private healthcare, these are often less decisive than they may initially appear and prohibiting private healthcare on egalitarian grounds is likely to run afoul of egalitarianism’s liberal commitments. There are several important theoretical hurdles to pass through for a successful egalitarian argument against private healthcare, which this paper aims to highlight.

## Egalitarianism and private healthcare

Egalitarians are generally critical of individuals using private healthcare in contexts where a comprehensive public healthcare system is the default. In countries such as Canada, Denmark, Finland, Iceland, New Zealand, Sweden and the United Kingdom, a comprehensive public healthcare system exists for all citizens, funded by general taxation and free at the point of use.^1^ Citizens are able to obtain all or most of their routine healthcare needs through this public system. This comprehensive public healthcare system is the *default*, from which one cannot opt out of paying taxes in favour of using private healthcare.^2^

Egalitarianism holds that equality, in some form, is a fundamental component of just institutions. This definition is ecumenical and deliberately ambivalent between distributive and relational accounts of egalitarianism. As Juliana Bidadanure and David Axelsen note, however, both distributive and relational egalitarianism are also liberal: “while equality is conceptualized as a chief value, a just society is also seen as one that protects an extensive set of liberal rights and provides opportunities for individuals to develop their own conception of the good life” (Bidadanure and Axelsen 2025). I follow Bidadanure and Axelsen in conceptualising egalitarianism as an inherently *liberal* egalitarian theory.^3^ By liberal, I simply mean a view which emphasises that the individual is the ultimate unit of moral consideration and that individual rights and freedoms constrain political action. Within this view is an acknowledgement of the value of personal choice and individual liberty, so long as this does not harm others. This is an ecumenical and widely accepted account within philosophy and bioethics, consistent with a wide range of theories of liberalism.

Egalitarianism is a prominent view within political philosophy, reflecting the ongoing dominance of John Rawls’ philosophical approach. It is also highly influential within bioethics and public health ethics, with theorists of health equity often drawing inspiration on concepts of equality and broader issues of egalitarian justice. This includes influential work on egalitarianism and health by theorists such as Norman Daniels, Ruth Faden, Charles Fried, Amy Gutman, Fabienne Peter, Madison Powers, and Jonathan Wolff, among others (Daniels 2007; Fried 1976; Powers and Faden 2006; Gutmann 1981; Wolff 2012). Henk ten Have and Maria do Céu Patrão Neves note that “egalitarian theory is an essential part of bioethics” (ten Have and Patrão Neves 2021, 451).

I use the term *private healthcare* as a shorthand phrase to refer to healthcare services and products provided outside the public healthcare system in exchange for financial payment by patients.^4^ Payment can be out-of-pocket (i.e. a patient paying a healthcare professional to perform a particular procedure) or mediated (i.e. a patient paying a premium to a private insurance scheme that then pays for their care when needed).

Private healthcare encompasses both *parallel* and *gap* private healthcare. *Parallel private healthcare* refers to the provision of services in the private sphere which are already available in the public healthcare system, but which can be provided privately with greater speed or at a higher quality. A paradigmatic example of parallel private healthcare is a patient with suspected cancer symptoms paying for expedited diagnostics or treatment. Someone might also pursue parallel private healthcare for better quality healthcare, either or both in terms of the quality of the healthcare services (such as paying for a more experienced surgeon) or the quality of the amenities (such as paying for private rooms or restaurant-quality meals in hospital).

*Gap private healthcare*, in contrast to parallel private healthcare, refers to the provision of healthcare services in the private sphere which are not available in the public healthcare system. A paradigmatic example of gap private healthcare is privately paying for a newly released patented drug not currently included in the public healthcare system’s list of services or paying for a cosmetic operation that the state does not fund.^5^

## The egalitarian case against private healthcare

Consider a pair of scenarios that motivate the egalitarian case against private healthcare:

***Alexandria:*** Alexandria is a poor single mother living in social housing. She has recently been diagnosed with cancer. The waiting time for her cancer operation on the public healthcare system is around 6 weeks.

***Brenda:*** Brenda is a wealthy and highly paid business executive. She has recently been diagnosed with cancer (the exact same type as Alexandria’s). She pays £50,000 to access a private clinic that can perform her operation within one week.

This scenario captures the *prima facie* egalitarian intuition many people have against private healthcare. It seems unfair to egalitarians that Brenda is able to access faster healthcare because of her socioeconomic status. This egalitarian objection is reflected in one form or another in public attitudes about the use of private healthcare in contexts where a comprehensive public healthcare system is the default. A survey conducted by a network of private healthcare providers in the UK showed that 24% of respondents “don’t approve of private healthcare in principle” (Independent Healthcare Providers Network 2024) and another recent survey showed that 55% of people opposed private healthcare on the grounds that it “undermines the NHS [National Health Service]” (Independent Healthcare Providers Network 2025). This principled opposition to private healthcare, generally on egalitarian grounds, is pervasive in societies with a comprehensive public healthcare system as the default, with public attitudes and rhetoric pointing this way (Hill 2025; Campbell 2024).

Egalitarians’ objection to private healthcare is not primarily about the fact that healthcare is *private*, but that it is *inegalitarian* and hence unfair.^6^ A system where private companies hold government contracts to deliver public healthcare, but where access is available to all on equal terms need not necessarily disturb egalitarians. Such a model (often referred to as the Bismarck Model) is consistent with egalitarian access and is used in many parts of continental Europe.^7^

Philosophers such as Norman Daniels, Amy Gutmann, Charles Fried, and Jonathan Wolff have addressed the general issue of egalitarianism and equal access in healthcare (Daniels 1985, 2007; Gutmann 1981; Fried 1976; Wolff 2012). The specific issue of accessing private healthcare in the context of a comprehensive public healthcare system, however, has received relatively little attention in the philosophical literature, despite its broad implications for our dominant (liberal) egalitarian framework of justice.^8^ On the one hand, private healthcare may promote autonomy by allowing individuals to pursue their conception of the good in the realm of health beyond what a liberal state can legitimately compel others to contribute towards. On the other hand, private healthcare may undermine the public healthcare system and harm those who must rely on that system, and it may involve *queue-jumping* in a way that symbolises social inequality.

Private healthcare is of particular concern to egalitarians in a way it may not be for rival distributive accounts such as sufficientarianism or prioritarianism. Sufficientarians focus on providing a core bundle of healthcare to all (negative thesis) but also argue that either we do not need to focus on distribution above the threshold (positive thesis) or that distributions above the threshold have lower moral priority (shift thesis) (Shields 2012; Casal 2007). As long as the public healthcare system provides an adequate bundle accessible to all, the fact that those above the threshold are able to access more or better healthcare is not obviously objectionable from a sufficientarian standpoint. Prioritarians focus on the worst off in terms of health, and these are generally accounted for by the public system’s focus on a core bundle of basic healthcare services. My claim is not that there are no prioritarian or sufficientarian arguments against private healthcare, but merely that they are not as ostensibly committed to opposing private healthcare as egalitarians are. Indeed, Benjmain Krohmal and Ezekial Emanuel’s arguments in favour of permitting private healthcare is based on the view that we should adopt a sufficientarian account of healthcare justice (where the public tier is good enough for everyone) instead of an egalitarian one (where the rich cannot purchase further healthcare above the publicly available minimum) (Emanuel and Krohmal 2009).

Despite the powerful egalitarian intuition that we feel about the scenario with Alexandria and Brenda, however, we need to interrogate these intuitions further. My strategy in the remainder of the paper is to explore various strands of the egalitarian case against private healthcare and evaluate whether they provide a convincing argument against it. First, I explore the argument that egalitarians should oppose private healthcare because it allows the affluent to buy health advantage and that this is objectionable on egalitarian grounds. Second, I analyse the argument that private healthcare harms disadvantaged groups who rely on the public healthcare system by diverting healthcare professionals from the public into the private sector. Third, and finally, I explore the view that egalitarians should oppose private healthcare on expressive and relational egalitarian grounds.

## Unjust access to health advantage

One of the strongest egalitarian intuitions about Brenda’s actions in the above scenario is that her wealth allows her to access health advantage not available to poorer people, and this is unfair. The argument that private healthcare amounts to individuals accessing unjust health advantage consists of two strands. The first strand is a simple complaint of horizontal fairness. It seems unfair to treat Brenda differently to Alexandria for the same health need when an egalitarian healthcare system should respond only to health need rather than other considerations such as ability to pay. Bernard Williams expresses this view, when he notes that healthcare should be sensitive only to need rather than wealth (Williams 2005).

The second strand is the concern that unjust advantage in one realm (such as wealth) can allow one to buy advantage in the domain of health, thus magnifying the injustice. Brenda may have benefited from brute luck features or an unjust basic structure that allowed her to become rich in the first place, which she now uses to translate into health advantage. Alexandria, on the other hand, suffers from injustice that makes life difficult for her as a poor single mother and is unable to access private healthcare. Brenda’s ability to access private healthcare combined with Alexandria’s inability to do so compounds the inequality of their starting disadvantage, which are themselves the product of injustice.

Harry Brighouse and Adam Swift have argued that health is a *latent positional good* (Brighouse and Swift 2006). Health is not *manifestly positional* (unlike education), but it is still positional. It matters not just how much health one has in absolute terms, but how much health one has relative to others. If health is at least partly positional in this sense, then private healthcare further enables unjust socioeconomic status to be translated into health advantage, with the health advantage giving one positional advantage in society. For example, a person healthier than us will tend to be more employable and therefore become better off than us financially. These egalitarian intuitions are very powerful, but a few considerations need to be kept in mind that may challenge how decisive this initial reaction is.

### Private healthcare is not necessarily just for the wealthy

Our egalitarian intuitions about Alexandria and Brenda may be influenced by the fact that one is a highly paid business executive while the other is a poor single mother. However, private healthcare is not necessarily only for the wealthy in our real world. Consider a variation of Alexandria from our earlier scenario:

Alexandria*: As before, but instead of waiting 6 weeks for her operation on the public healthcare system, Alexandria goes cap in hand and cobbles together funds from various sources (family, friends, crowdfunding, and all her personal savings) to fund her operation through the private system.

It is possible that our intuitions in Alexandria* will be different from Alexandria. There may still be a complaint of horizontal unfairness, but the concern around the wealthy being able to translate their economic advantage into health advantage is reduced in such scenarios. Alexandria is not wealthy and does not translate her *wealth* into advantage in the realm of health.

A large proportion of people who use private healthcare in the context we are concerned with also do so through insurance policies funded through modest premiums. Consider another variation of our earlier scenario:

Brenda*: As before, but Brenda has a health insurance policy with modest premiums of £50 per month which she contributes towards. She now makes a claim on this policy to fund her expedited cancer operation.

Brenda* seems different to Brenda. One simple reason for this is that while very few people can afford to pay £50,000 for a private operation, many more people can afford to pay £50 a month for an insurance policy that would provide them with such care if and when the need arises. A luck egalitarian may point out that egalitarians have no reason to object to Brenda’s prudence in this case. If Alexandria prefers to put £50 towards her holiday fund instead of a health insurance policy that would enable her to access expedited private healthcare if the need arises, then she has no grounds for complaint if she is unable to access private healthcare while others are able to. Alexandria has made a calculated gamble, and it is simply bad option luck that she is unable to access private healthcare now that she has become ill (Knight 2013; Stemplowska 2009). The luck egalitarian response may not satisfy all egalitarians.

Egalitarians can respond that even if there are cases where people like Alexandria can access private healthcare through crowdfunding and insurance, they are still systematically disadvantaged compared to people like Brenda. While crowdfunding and insurance are options, they are not necessarily available to everyone. £50 a month for a poor, single mother may still be a significant amount. The insurance premium could be a significant portion of the household grocery bill or electricity expenses, for example, so the fact that insurance premiums are relatively affordable may not fully allay egalitarians’ concerns about people in Brenda’s position and there may be some who are unable to afford even this modest amount. Crowdfunding is also an unrealistic expectation for most people, with the success of such campaigns heavily dependent on social networks and the ability to generate media attention – factors that are themselves linked with social advantage. For example, crowdfunding campaigns are much more likely to succeed for people who are younger, white, and male than for minority ethnic, older or female patients (Davis et al. 2023; Zhang et al. 2025). The fact that people like Brenda have to choose between holidays and healthcare while people like Alexandria do not will also not satisfy many egalitarians.^9^

The plausibility of the luck egalitarian response depends partly on the existence of a fair starting point, so that decisions reflect outcomes of what Ronald Dworkin calls option luck rather than brute luck (Dworkin 1981). Given background injustice and the lack of a fair starting point for everyone, individual exercises of choice to purchase private healthcare (and even private insurance) may not be fully just and would simply reflect this unjust starting point. This point, however, implies that there is at least a hypothetical state of affairs in which private healthcare would not be unjust from an egalitarian standpoint. If everyone started from an equal starting point, then the fact that some people choose to spend money on private healthcare would not be objectionable.

The only way egalitarians can remain opposed to private healthcare in situations where everyone started from an equal starting point would be to believe that healthcare is its own unique sphere that ought to be governed by its own distributive principles. This view is held by theorists such as Michael Sandel and Michael Walzer (Walzer 1983; Sandel 2012). It also relates to the distinction James Tobin has drawn between *specific* and *general* egalitarianism (Tobin 1970). General egalitarianism focuses on egalitarianism at a general level (general taxation being one general egalitarian policy example) while specific egalitarianism cares about the egalitarian distribution of specific goods and bundles independently of the general distribution. The issue with specific egalitarianism in the case of healthcare is that it risks being unduly perfectionist, given that reasonable people disagree about how much they care about health and healthcare relative to other pursuits. This links into the next consideration egalitarians need to keep in mind, and arguably one that looms the largest, in considering the initial objections to the scenario.

### Liberalism and autonomy

The liberal consideration may challenge our egalitarian intuitions about private healthcare even if we do not assume background justice. Even without an egalitarian starting point, citizens in liberal societies make decisions about how to spend their money without these decisions being the subject of external interference or egalitarian opposition. If we allow people (including those without much money) to spend their money on holidays for their family or iPhones for themselves, why can they not spend that same amount of money for private medical treatment? The challenge becomes more acute when we reflect on the nature of a public healthcare system.

A public healthcare system will provide a bundle a services accessible to all. What is included in this bundle is a function of several things, including how much it would cost to provide. The budget that funds the healthcare system depends on citizens’ tax contributions, and a legitimate state must be able to justify to its citizens the level of tax they are being charged and what it is going towards. A public healthcare system therefore walks a tight line between providing enough and good enough healthcare services for all while not demanding too much of its citizens – many of whom may prefer a state that cares less about health and gives them more post-tax income to spend on their own leisure activities. Different reasonable conceptions of the good life will also disagree on how much public money should be spent on hospitals and healthcare compared to, for example, public transport or education.

The state, reflecting the popular will, may take a view that Treatment A should be provided in the public bundle while Treatment B should not be. For example, the state decides that coronary bypass operations should be publicly funded but not some cosmetic operations. Insofar as egalitarians are also liberals, they may not be able to object to a person purchasing *gap private healthcare* (such as cosmetic surgery) to cover what the state could not legitimately provide on the public system, on pain of undue perfectionism (Rawls 1993). The same general view may hold for *parallel private healthcare*. The state could significantly increase the amount it spends on healthcare, in an effort to reduce waiting lists and make services quicker. However, this would carry significant opportunity costs and mean that other valuable social goods would have to be sacrificed. Some citizens will prefer that the state spends more money on non-healthcare-related goods while others will prefer that the state spends more on healthcare to make waiting lists shorter. Those who would have preferred that the state spend more on healthcare (and would have been happy to pay more in tax towards this) may thus decide to spend their money on parallel private healthcare. An egalitarian, insofar as they are also liberal, may have no complaint against such actions.

Furthermore, private healthcare may be purchased not for health-related reasons, but for reasons of convenience and comfort. For example, some patients would prefer to pay for a private room in a hospital and have access to restaurant-quality meals in the ward. The median voter may not want such services provided with their taxes, but it is harder to see how the liberal state could legitimately restrict patients from making such choices for themselves.

### Social determinants of health

A final consideration that may challenge our views about the first egalitarian line of argument against private healthcare is to consider empirical evidence about the social determinants of health. The social determinants of health refer to the non-medical factors that influence health: income, housing, education, transport, social capital, and so on. Research by social epidemiologists over the past few decades has shown that healthcare has a comparatively minimal impact on health outcomes compared to the influence of the social determinants of health (Commission for the Social Determinants of Health 2008; Marmot and Wilkinson 2006; WHO 2021). The social determinants of health have a significantly greater impact on health outcomes. Two observations arise from this empirical background.

Firstly, the egalitarian intuition that Brenda is buying health advantage might be blunted if healthcare’s impact on health is shown to be limited. If evidence from the social determinants of health is correct, then it would seem that private *healthcare* has a relatively limited impact on health. However, this view overlooks the fact that healthcare has a salient impact on *individuals*’ health, even if its aggregate impact on population health is not significant relative to the social determinants of health (Go 2023).

Secondly, the social determinants of health could be used as a *reductio ad absurdum* argument against the egalitarian intuition against private healthcare. Richer people can pay to live in areas with lower levels of air pollution, more green areas and better access to fitness facilities. Income levels themselves have a direct impact on health outcomes, including reducing levels of psychological stress associated with employment and precarity. Given that certain people can make health-promoting decisions in this way with equivalent if not greater impact on their health, opposing private healthcare may also commit the egalitarian to opposing actions such as buying a house in a less polluted area. This should strike egalitarians as unduly interventionist and contrary to core liberal commitments, and it may mean that the initial intuition against private healthcare may need further consideration for reasons of consistency.

The egalitarian argument that private healthcare should be opposed on the grounds that it allows the wealthy to unfairly buy health advantage is an intuitive and appealing argument, but it faces at least three hurdles outlined above: private healthcare is not necessarily just for the wealthy; the commitment to liberalism entails certain freedoms and prerogatives to spend one’s money as one chooses, especially given that the state cannot devote as much to healthcare as some citizens may want; and consistency may require opponents of private healthcare to also oppose actions that some citizens take to improve their health in other, non-healthcare related ways (which is implausible).

## Harm to patients in the public healthcare system

Another core argument against private healthcare that many egalitarians advance is that using private healthcare causes harm to patients in the public healthcare system because it diverts healthcare professionals away from the public sector into the private sector. Because the supply of doctors is limited and inelastic,^10^ those who work in the private and public sectors come from the same pool (Walpole 2023). In New Zealand and the UK, for instance, most doctors work for the public healthcare system while maintaining part-time private practices. This means that time spent working in the private system comes at the expense of time in the public system (Flood and Xavier 2008; Fenton 2011).

Because doctors practising in the private healthcare sector tend to have a lower caseload and see fewer patients per day than they would in the public system, the *total supply* of appointment slots is reduced.^11^ Doctors who work simultaneously in private and public practice have few incentives to reduce waiting times in the public system since long waiting times are a motivating factor for patients to seek private healthcare and in the interests of doctors with private practices (Robbins 2023).^12^ Patients who use private healthcare contribute towards these incentive structures for doctors that harm patients who rely upon the public healthcare system.

This is not a distinctly egalitarian objection as the harm-based argument could be used by non-egalitarians, but it has an egalitarian flavour if we assume that those left behind in the public healthcare system are those who are the most disadvantaged. This objection depends primarily on the empirical evidence, and there are good reasons to believe that private healthcare may come with certain supply-side effects that are also to the benefit of the least well-off.

Firstly, one may argue that a thriving private healthcare system has important benefits in terms of increasing the total supply of healthcare resources and incentivising a greater number of people to enter the healthcare profession, knowing they can supplement their income through private practice. There is strong evidence in the literature around financial incentives and their role in recruiting and retaining medical professionals (El Koussa et al. 2016). A person who would otherwise not train to become a doctor, knowing the low rate of pay in the public system, may be tempted to enter the profession if they know that they can supplement their income with private sector work. Over time, this will mean an increase in the supply of doctors and appointment slots, to the benefit of all.

Secondly, existing doctors may increase the total *hours* they work if they can treat lucrative patients, and this increases the total supply of appointment slots in the health sector. For example, writing in the US Medicare context, Jeffrey Clemens and Joshua Gottlieb note that a 2% increase in payments to doctors resulted in a 3% increase in the amount of medical care provided (Clemens and Gottlieb 2014). Patients who seek private healthcare, in such situations, would incentivise doctors to increase their hours in the private sector without reducing their work in the public sector, and this therefore reduces demand on the public system. This appears to be a Pareto-efficient outcome and benefits those on the public waiting list too.

Thirdly, an argument based on John Rawls’ *difference principle* may be available. The *difference principle* argues that inequalities can be justified when these are to the advantage of the least well-off (Rawls 1971). If our empirical conjecture is correct that there are supply-side benefits to private healthcare which are to the benefit of all (including the least advantaged) even if they result in inequality, then egalitarians may have a principled reason to not oppose such an arrangement. Consider a case from 2008, when the Royal Bank of Scotland donated a state-of-the-art CT machine to an Edinburgh public hospital with the proviso that 25% of its diagnostic capacity be reserved for employees and members of the bank’s healthcare scheme (The Herald 2008). The difference principle provides a principled reason for egalitarians to accept (or at least tolerate) this arrangement. The case of private healthcare may be analogous, where those who pay can get expedited or additional access to healthcare services, but where their actions also bring about benefits to the worst off. There may be other egalitarian objections to the arrangement, including based on relational considerations (as I turn to shortly), but insofar as private healthcare may be to the advantage of the least well-off, the complaint of harm is defused.^13^

## Relational inequality, queue-jumping, and abandoning fellow citizens

Egalitarians may object to private healthcare on relational grounds rather than distributive concerns around harm or the unfair distribution of health advantage. Relational egalitarianism is the view that what matters from an egalitarian standpoint is not the distribution of material goods or the neutralising of luck between individuals, but how they stand in relation to one another (Anderson 1999). Relational equality obtains when there is an absence of domination and status hierarchy, and people instead stand alongside each other as social equals (Schemmel 2021).

An important relational egalitarian argument against healthcare links in well with the harm-based argument explored earlier, noting concerns about reduced incentives for improving the public healthcare system. The availability of a private healthcare system may isolate users of the private system from caring about the public system and its users. Those with the most influence and power tend to be those who can opt out of the public system and seek care in the private sector, with the result that they have little stake in the performance of the public system.^14^ A system that allows those with the means from exiting the public healthcare system removes any personal interest one might have in improving that system to the detriment of those who remain in it. Users of private healthcare may come to see the public system as irrelevant to them and stop caring about its improvement politically, isolating themselves politically and relationally from the public healthcare system and its users.

A critic of this argument might object that people will still have a stake in the public healthcare system because some services – accident and emergency departments and intensive care services being the most obvious candidates – are only available in the public system. This is indeed the case in most comprehensive public healthcare systems supplemented by a smaller private healthcare sector (White 2009). The UK’s private healthcare sector, for example, does not generally have the capabilities to perform highly complex surgeries or to manage rare conditions, nor do they have intensive care and emergency services. These services are available only in the public sector.^15^

However, this consideration is not enough to mitigate the general effects of the private healthcare system on the public system. Emergency departments and intensive care units comprise only a small part of the public healthcare system. Someone having a stake in a small part of the public system does not necessarily mean that they will be sufficiently motivated to push for improvements and reforms in areas of the healthcare system that provide services they can access in the private system, such as issues relating to elective surgery waiting lists or cancer care. It may even perversely incentivise such people to unjustifiably support the diversion of funding from other areas into the sphere of the public healthcare system upon which they have to rely.

The core of the relational egalitarian objection to private healthcare is that it violates norms of relational equality between citizens. Using private healthcare, on this view, may express disdain to those on the public healthcare system. It may imply that citizens are not in the same boat, with private healthcare being a form of queue-jumping that expresses a disdain for the health needs of those waiting on the public healthcare system (Go 2026). The wrong of private healthcare could also lie in its expressive dimension, with such a practice failing to express equal concern and respect to fellow citizens (Go 2023). This is a powerful argument, but a series of further considerations should be borne in mind.

Firstly, relational egalitarian theories such as Elizabeth Anderson’s *democratic equality* are more appropriately characterised as sufficientarian rather than egalitarian in nature (Schemmel 2011). In her writings on private schools, Anderson argues that above the sufficiency threshold of educational quality that all schools should provide, parents may pay for their children to have access to more elite level schooling: “Parents who want to provide their children with more education than the minimal required to enable them to complete successfully a serious four-year college degree are free to do so, using their own private resources” (Anderson 2007, 615). Anderson further notes that “the consequence of implementing an equality-of-resources criterion of fair educational opportunity would be to level down opportunities to the tastes of the median voter” (Anderson 2007, 615).

Insofar as private education is analogous to private healthcare from a relational egalitarian standpoint, then Anderson’s arguments show that relational egalitarianism need not necessarily oppose private healthcare. Anderson’s arguments are that relational egalitarians ought to care about social relations instead of the distribution of resources, such as education. For Anderson, the wealthy being able to purchase additional services on top of the guaranteed social minimum need not run contrary to relational egalitarian norms. However, other relational egalitarians such as Christian Schemmel argue that relational egalitarians should care about distribution much more than Anderson suggests (Schemmel 2011). This includes paying attention to distributions in the domain of health and healthcare (Schemmel 2021).

Schemmel makes some comments about healthcare, including his statement that “[a] relational egalitarian society has to institute a universal healthcare system” (Schemmel 2021, 277). The core of Schemmel’s arguments about relational egalitarianism in health, however, focus on health inequality (viewed in terms of health outcomes) rather than access to healthcare as such. This makes Schemmel’s arguments prone to the observations noted earlier in Sect. 3 about the social determinants of health. If our focus is on health inequality, then private healthcare may be neither here nor there since the social determinants of health are the primary influence on health outcomes and not healthcare.

Schemmel is aware of this implication:

[If] it turns out that more equal opportunity to buy things (including healthcare) is not what makes people healthier in more egalitarian societies (as many public health researchers indeed argue) then the support generated by the finding that the degree of equality demanded by this conception also makes for (roughly) equal health would be weak (Schemmel 2021, 269).

Schemmel’s relational egalitarian argument, then, is also contingent on empirical evidence around the social determinants of health in the same way as other egalitarian theories. If public health researchers and social epidemiologists are correct that healthcare is not the main determinant of health outcomes, then egalitarians’ preoccupation with private *healthcare* may be a red herring and detract from the true target of health justice. The observation that healthcare can have a significant effect on an *individual* patient in need is consistent with the claim that the social determinants of health have a comparatively bigger effect on population health outcomes (and therefore on health justice).

Secondly, even if there are strong relational egalitarian arguments against private healthcare, it remains an open question how much weight should be given to expressive and relational harms when balanced against liberal commitments to spend one’s resources as one chooses and agent-relative prerogatives to look after our own interests and health. The fact that purchasing private healthcare would express a form of inequality, or that accessing such care connotates the view that citizens are not in the same boat, does not provide an all-things-considered argument against private healthcare. The relational egalitarian objection against private healthcare, plausible though it is, needs to be supplemented with and balanced against other considerations and arguments.

## Conclusion

This paper has explored the question of whether egalitarians must oppose private healthcare. The aim of the paper is not to come to an all-things-considered view about whether egalitarians must oppose private healthcare, but to show the argumentative hurdles that need to be met for such a position. While there are strong egalitarian arguments against private healthcare based on harm, unfairness, and relational inequality, there are equally strong arguments grounded in liberal autonomy and the implications of the social determinants of health that need to be kept in mind. It is therefore far from clear that egalitarians must oppose private healthcare.

## Footnotes

1 This is often called the Beveridge Model of healthcare.

2 In this context, I am not concerned with (for example) the Australian Medicare system, which is a rare example of a mixed public–private system with tax credits available to high-income earners who opt out of using the public system.

3 Those who disagree with this conceptualisation of egalitarianism can simply reframe the question in the title of this paper as whether *liberal* egalitarians ought to oppose private healthcare.

4 I therefore use the terms ‘purchasing’, ‘seeking’ or ‘accessing’ private healthcare interchangeably.

5 Cosmetic surgery still meets the criteria for healthcare. Many of these operations meet a clear medical need, such as plastic surgery for burns patients or cosmetic breast reconstruction after mastectomy (Go 2018). In any case, even if they are purely elective and non-medically necessary, these operations are carried out by medical professionals in a clinical setting and arguably meet the standard for healthcare.

6 Analogously, in his arguments against private schools, Adam Swift notes that his objection is not about the *privateness* but the *unfairness* of such schools (Swift 2004, 9).

7 Opponents of privatisation in general might nevertheless object. See, for example, Walzer (1983), Cordelli (2020), Sandel (2012), and Dorfman and Harel (2016).

8 Notable exceptions include Holm (1989), Weale and Clark (2010), Fourie (2016), and Emanuel and Krohmal (2009).

9 Holidays could be a matter of justice, insofar as discretionary time and leisure may be an appropriate distribuendum of justice (Rose 2016).

10 This is so even if we consider the problem of brain drain and the foreign movement of medical labour. The *global* supply of doctors remains relatively inelastic, and this is the relevant fact. If the supply of doctors increases in rich countries through healthcare workers’ migration, this generally means a reduction in supply of doctors in poorer countries, to the detriment of people in these countries. See, for example, Brock and Blake (2015).

11 Indeed, the reduced caseload and additional contact time between each patient is often emphasised in marketing around private healthcare (St John and St Elizabeth Hospital 2024).

12 In a notable case, the Ophthalmological Society of New Zealand tried to block the entry of more ophthalmologists because it would lower the public waiting times and thereby reduce demand for their members’ private practices (Commerce Commission v Ophthalmological Society of New Zealand & Ors 2004).

13 I assume a counterfactual notion of harm, where harm requires that one be made worse off than one otherwise would be.

14 Albert Hirschman observes this phenomenon when parents move their children to private schools (Hirschman 1970, 51–52).

15 Critics of private healthcare note that the private sector leaves these services (which come with high overhead costs) almost entirely to the public system and focus on more profitable services – a phenomenon known as ‘cream-skimming’.
